# H1N1pdm09 returns: a comparative study in Anhui, China

**DOI:** 10.1128/spectrum.02218-24

**Published:** 2025-09-08

**Authors:** Si-Tian Yang, Min-Hao Hu, Wei-Xi Fang, Hui-Min Diao, Harry Asena Musonye, Ji-Xiang Huang, Yi-Dan Xia, Jun-Ling Yu, Lei Gong, Sai Hou, De-Xi Li, Wan-Rong Luo, Xue Zhou, Xian-Wei Luo, Jia-Bing Wu, Hai-Feng Pan, Jun He

**Affiliations:** 1 Anhui Provincial Center for Disease Control and Prevention, Hefei, China; 2 Tangshan Center for Disease Control and Prevention 176762 , Tangshan, China; 3 School of Public Health, Anhui Medical University 569061, Hefei, Anhui, China; 4 Anhui Provincial Academy of Preventive Medicine, Hefei, Anhui, China; 5 Anhui Academy of Medical Sciences, Hefei, China; 6 Inflammation and Immune Mediated Diseases Laboratory of Anhui Province, Anhui Medical University, Hefei, Anhui, China; 7 Department of Epidemiology and Biostatistics, School of Public Health, Anhui Medical University 569062, Hefei, Anhui, China; Victorian Infectious Diseases Reference Laboratory, Melbourne, Australia

**Keywords:** epidemiology of influenza, H1N1pdm09 outbreak, HA gene mutations, cross-immunity protection, control strategy

## Abstract

The 2022/2023 season witnessed a rapid resurgence of H1N1pdm09 in Anhui Province, China, surpassing previous years, prompting an examination of hemagglutinin (HA) gene mutations and cross-immunity in this study. Anhui Province’s surveillance data established the detection threshold for H1N1pdm09 using the Moving Epidemic Method. Joinpoint regression compared weekly percent change (WPC) rates. A total of 103 strains of H1N1pdm09 were sequenced and analyzed, and cross-reactivity was assessed using *P*_epitope_ model. The ILI% (percentage of outpatient visits for influenza-like illness) and influenza detection rate for the 2022/2023 season were 6.82% and 31.90%, respectively, which surpassed those of 2017/2018–2021/2022. The H1N1pdm09 subtype dominated this outbreak. The epidemic threshold for H1N1pdm09 was 3.53%, exceeded in the sixth week of 2022/2023, leading to a shorter but intense epidemic phase. The WPC for the 2022/2023 season reached 376.40% (95% confidence interval: 253.4%–545.8%), faster than in the prior seasons. Sequence analysis showed that a majority of the 32 strains, obtained in the 2022/2023 season, belonged to clade 6B.1A.5a.2a, with only one strain identified as 6B.1A.5a.2a.1. Cross-protection efficacy against representative strains from the 2017/2018 and 2018/2019 seasons was limited (25.54%, 34.69%). This analysis provides insights into Anhui’s influenza situation, including early warning thresholds, molecular and immune perspectives, and cost-effective prevention and control strategies.

**IMPORTANCE:**

The H1N1pdm09-associated morbidity and mortality impact negatively on the socio-economic status of the affected population. Potential modification of the genetic and antigenic traits of the virus, as it circulates, has raised concern that it may hinder a host’s immune response or vaccine efficacy. These have prompted researchers to come up with intervention measures to manage H1N1pdm09 as a pathway to ease the pressure on public health. This analysis provides insights into Anhui’s influenza situation, including early warning thresholds, molecular and immune perspectives, and cost-effective prevention and control strategies.

## INTRODUCTION

On 11 June 2009, the World Health Organization (WHO) declared a global pandemic of H1N1 influenza. The latest H1N1 strain responsible for this outbreak was H1N1pdm09. Although the pandemic was officially declared over on August 11 of the following year (1), the virus has replaced the previous seasonal H1N1 strains and continues to circulate worldwide (2). The H1N1pdm09-associated morbidity and mortality impacts negatively on the socio-economic status of the affected population. Potential modification of the genetic and antigenic traits of the virus, as it circulates, has raised concern that it may hinder a host’s immune response or vaccine efficacy. These have prompted researchers to come up with intervention measures to manage H1N1pdm09 as a pathway to ease the pressure on public health. Some of the measures include surveillance, vaccination, and non-pharmaceutical interventions (NPIs).

The implementation and effect of NPIs (social distancing, isolation, travel restrictions, school closure, and wearing masks) can be prominently illustrated by the low circulation of influenza virus worldwide during the outbreak of coronavirus disease 2019 (COVID-19) compared to pre-pandemic times (3). For instance, China experienced the onset of the COVID-19 pandemic in December 2019 (4). In early 2020, there was a rapid increase in reported cases of SARS-CoV-2 infections globally. In April 2020, to mitigate the pandemic, China primarily adapted NPIs, which saw a drastic reduction in the COVID-19 cases. Similarly, various states in the United States also employed NPIs to mitigate the spread of the virus (5). Research indicates that the implementation of the non-pathogen specific NPIs led to a reduction of around 79% and 67% of influenza cases in China and the United States, respectively (5). Nonetheless, in February 2023, the H1N1pdm09 subtype of influenza made a resurgence, which has been attributed to the easing of COVID-19 NPIs measures. This shift in dynamics has raised fears that as the susceptible population grows, massive future H1N1pdm09 outbreaks may occur after all NPIs are withdrawn, putting more pressure on healthcare.

The efficacy of influenza vaccinations remains unsatisfactory. Although they can prevent most influenza viruses, the protective effectiveness of early vaccinations lasts only a year, and influenza cases usually peak a year later (6, 7). While universal influenza vaccines have the best possibility of giving long-term protection, their production timescale and universality are uncertain (8). Antigenic shift enables the creation of antigenically novel virus strains capable of causing pandemic outbreaks. Regardless of these challenges, there have been concerted efforts to modify how NPIs and vaccines strategies are employed in management of the H1N1pdm09 virus. One such effort is the adaptation of the *P*_epitope_ model and the Moving Epidemic Method (MEM) approaches to elucidate infection dynamics of influenza outbreak.

The H1N1pdm09 virus genome is composed of eight RNA segments (9). Among them, the five antigenic determinants: Ca1, Ca2, Cb, Sa, and Sb in the head of the hemagglutinin (HA) protein are crucial targets for recognition by the immune system and for antibody neutralization (10). The HA protein’s head region includes three structural elements: the 220 loop, the 130 loop, and the 190 helix. These elements are binding sites for the host’s sialic acid receptors (11). These sites are crucial for virus replication and infection and thus may determine the efficacy of H1N1pdm09 vaccines. In this regard, the *P*_epitope_ model has been developed to assess antigenic distances and vaccine efficacy. Studies have revealed that the model is more accurate than ferret antisera inhibition assays (12, 13). However, the lower vaccination rates in Anhui Province may not significantly impact the prevalence of influenza in the population. Nonetheless, the hypothesis that cross-immune responses generated by natural infections, similar to vaccination, can reduce the susceptibility of the population during subsequent outbreaks has been proposed (14). Therefore, utilizing the *P*_epitope_ model for evaluating the effectiveness of cross-immune responses may be cost-effective and accurate.

The implementation of NPIs relies on a precise prediction system. Setting alert thresholds for specific regions can promptly alert authorities and the public to the onset of influenza and enable them to take necessary preventive measures (15). In view of this, the WHO has put forth several predictive and alert methods, such as the MEM. This method has been widely utilized in the European region due to its exceptional performance and practicality (16–19). In contrast, its application in China has been limited. As a threshold-setting method, MEM lacks detailed prediction of the increasing or decreasing trends in detection rates during the monitoring process. However, combining this quantified trend with quantified thresholds will help make our predictions for the next year more reasonable. Joinpoint regression, a method known for its rigorous approach, employs time-series data to identify changes within trends (20). Although previous studies have employed joinpoint regression to research on the changing trends of influenza virus, most of these studies have focused on macro-level analysis across multiple years (21, 22). There has been a dearth of studies quantifying the changing trends of specific influenza subtypes within a single year. Understanding the propagation rates of different subtypes and their annual variations among populations holds significant potential for devising targeted preventive and control measures tailored to the unique characteristics of each subtype.

In this study, we adopt a comparative research approach. Leveraging the MEM, we establish an alert threshold for the H1N1pdm09 subtype of influenza in Anhui Province. Employing joinpoint regression, we elucidate the current trend and changes in the circulation of the A(H1N1) subtype. We quantitatively assess the weekly percent change (WPC) in detection rates. Additionally, through comparative analysis of H1N1pdm09 subtype genomic data spanning from 2017/2018 to 2022/2023, we identify viral antigenic drift characteristics. By synthesizing these findings, we aim to comprehensively elucidate the fundamental causes behind the onset of a new wave of influenza.

## MATERIALS AND METHODS

### Source of data

Data on influenza-like illness (ILI) and laboratory-confirmed influenza cases in Anhui province, collected at the laboratories of 16 municipal Centers for Disease Control and Prevention (CDC) as well as 24 sentinel hospitals, were retrieved from the “China Influenza Surveillance Information System”. The laboratories and the sentinel hospitals are characterized with significant patient volumes and are situated in densely populated areas across the province.

### Solation and identification of H1N1pdm09 strains

The H1N1pdm09 strains were isolated and identified in accordance with the Chinese Influenza Surveillance Protocol (23). Briefly, nasopharyngeal swab specimens were collected from ILI patients who had not taken antiviral medications within three days of symptom onset at sentinel hospitals. The specimens were preserved in the virus sampling tubes (Yocon, MT0301-1) and transported at 4°C to municipal-level influenza surveillance network laboratories for reverse transcription polymerase chain reaction (RT-PCR) identification. Positive specimens were subsequently subjected to chicken embryo and MDCK (Madin-Darby Canine Kidney) cell culture, followed by HA test and hemagglutination inhibition (HI) test at the provincial-level influenza reference center to determine the viral titer and subtype.

### Virus sequencing and sequence analysis

In this study, a total of 103 H1N1pdm09 viral strains were selected and isolated from MDCK cells for sequencing across the years 2017/2018 to 2022/2023 based on urban and annual prevalence. Nucleic acid extraction was performed according to the instructions of the nucleic acid purification kit (Tianlong Technology, Lot: 21021210T324). Following nucleic acid extraction, the complete genome of the influenza virus was amplified using the SuperScript III One-Step RT-PCR System with Platinum Taq High Fidelity (Thermo Fisher, 12574035), and RT-PCR detection was carried out as per the manual. Primers and probes were designed in accordance with recommended sequences from the National Influenza Center at the Chinese Center for Disease Control and Prevention (Table 1). The PCR extraction products were verified by gel electrophoresis and purified using the QIAquick 96 PCR Purification Kit (QIAGEN, 28183). The PCR extraction products were quantified by Qubit dsDNA HS Assay Kit (Invitrogen, Q32851), and library preparation was performed using the MGIEasy Respiratory Microbiome Genome Amplification Kit (MGI, 940-000059-00) and the MGIEasy Rapid PCR-Free FS DNA Library Prep Kit (MGI, 940-000019-00) through multiplex PCR amplification. Sequencing was conducted on the MGISEQ-2000RS sequencer using the following parameters: read 1 100 bp, read 2 100 bp, and dual barcodes of 10 bp each. The raw sequencing reads were filtered, identified, and subjected to genomic assembly as follows: *de novo* assembly without a reference was performed separately using Velvet (v1.2.10) and Edena (v3) software (with a minimum contig length of 200 bp). Simultaneously, reference-based assembly was carried out using BWA (v0.7.17) and SAMtools (v1.8) in combination with reference sequences. All assembly results were then integrated and polished using Clean Reads, ultimately yielding the final assembled genome. Final sequences were assembled and annotated using the MGI FluTrack software (MGI, 970-000225-00). The H1N1pdm09 genomic sequences generated in this study have been deposited in the Global Initiative on Sharing All Influenza Data (GISAID) database under the accession EPI2913321–EPI2914296 and EPI2914812–EPI2914819.

**TABLE 1:** Primer sequence for multisegment

Primer names	Primer sequence
Uni-12/Inf1 (primer A)	5′-GGGGGGAGCAAAAGCAGG-3′
Uni-12/Inf3 (primer B)	5′-GGGGGGAGCGAAAGCAGG-3′
Uni-13/Inf1 (primer C)	5′-CGGGTTATTAGTAGAAACAAGG-3′

After obtaining the genomic sequences, the HA segment of the reference strains was downloaded from the GISAID database. Average genetic distances were calculated using MEGA 6.0. Multiple sequence alignments of the HA gene were performed using MAFFT v7.505 (24). Systematic phylogenetic reconstruction based on maximum likelihood was carried out using IQ-TREE v2.2.0 (25) with 1,000 bootstrap replicates. The resulting trees were visualized using iTOL (https://itol.embl.de/). Amino acid analyzes were conducted using R software v4.2.2. Glycosylation site predictions were made using NetNGlyc 1.0 (https://services.healthtech.dtu.dk/services/NetNGlyc-1.0/) with a threshold of 0.5. Amino acid positions in this study followed the H1 numbering convention. The three-dimensional structure of A/Michigan/45/2015 was obtained from (https://swissmodel.expasy.org/, SMTL ID: 7kna.1), and HA protein visualizations were created using Pymol.

### Statistical analyses

#### Descriptive statistics

ILI and influenza positivity rate descriptions were delineated based on the influenza surveillance year, spanning from April 1st of the current year to March 31st of the following year (26). The annual influenza epidemic intensity was divided into the period from the 43rd week of the current year to the 42nd week of the following year, following the characteristics of the MEM model application with a single peak. The IBM SPSS software 23.0 was used for normality testing and descriptive statistics. Differences in rates among groups were assessed using the Pearson *χ*^2^ test (*P* < 0.05 indicating statistical significance).

### Estimating epidemic threshold and intensity using MEM and joinpoint regression

For the qualitative classification of epidemic intensity, a MEM model was established using H1N1pdm09 subtype positivity rate data from six active influenza seasons (2012/2013–2013/2014, 2015/2016–2018/2019). The epidemic thresholds for both pre-epidemic and post-epidemic phases were calculated as the upper limit of 95% one-sided confidence interval of H1N1pdm09 subtype positivity proportion for the highest 30 weeks before (after) the peak from the six influenza seasons. The upper limits of the 45%, 90%, and 97.5% one-sided confidence intervals of the geometric mean of the highest weekly influenza positivity proportions from these six influenza seasons were defined as the thresholds for moderate, high, and very high intensity, respectively. A detailed operational procedure of the MEM is outlined in Vega’s study (18). The data calculations for MEM were performed using R software (version 4.2.2).

For the quantitative analysis of epidemic intensity, the Joinpoint Regression (version 5.0, the National Cancer Institute) was employed for segmented regression analysis. The choice of model category was based on the weekly detection rate data set. If the data followed a normal or approximately normal distribution, a linear data model was selected. If the data followed an exponential or Poisson distribution, a logarithmic linear data model was chosen (27). The inflection point analysis and parameter estimation were conducted using grid search method, with the optimal model identified through the Weighted Bayesian Information Criterion method. This process facilitated the determination of the WPC along with its 95% confidence interval. A *t*-test was employed to assess the statistical significance of WPC changes, whether they were decreasing or increasing.

### Establishing cross-protection level efficacy for A(H1N1) pdm09 strain using *P*_epitope_ model

The antigenic distance is the proportion of differences in amino acids on the antigenic *epitope* clusters between two viral strains. Among the five *epitope*s on the HA protein, the *epitope* with the highest proportion of differences was defined as the “*P*_epitope_” (28). Since the cross-protection effect is linearly related to the antigenic distance between viral strains (28), the *P*_epitope_ model was employed to estimate the cross-immune protection effect of H1N1pdm09. The parameter was defined as *E* = −1.19 × *P*_epitope_ +0.53 (29), and the implementation method followed the approach outlined in Bonomo’s study (13). At *P*_epitope_ equals zero, the cross-protection efficacy was scored as 53.00%.

## RESULTS

### Basic overview of influenza for the years 2017/2018–2022/2023

**Fig 1:**
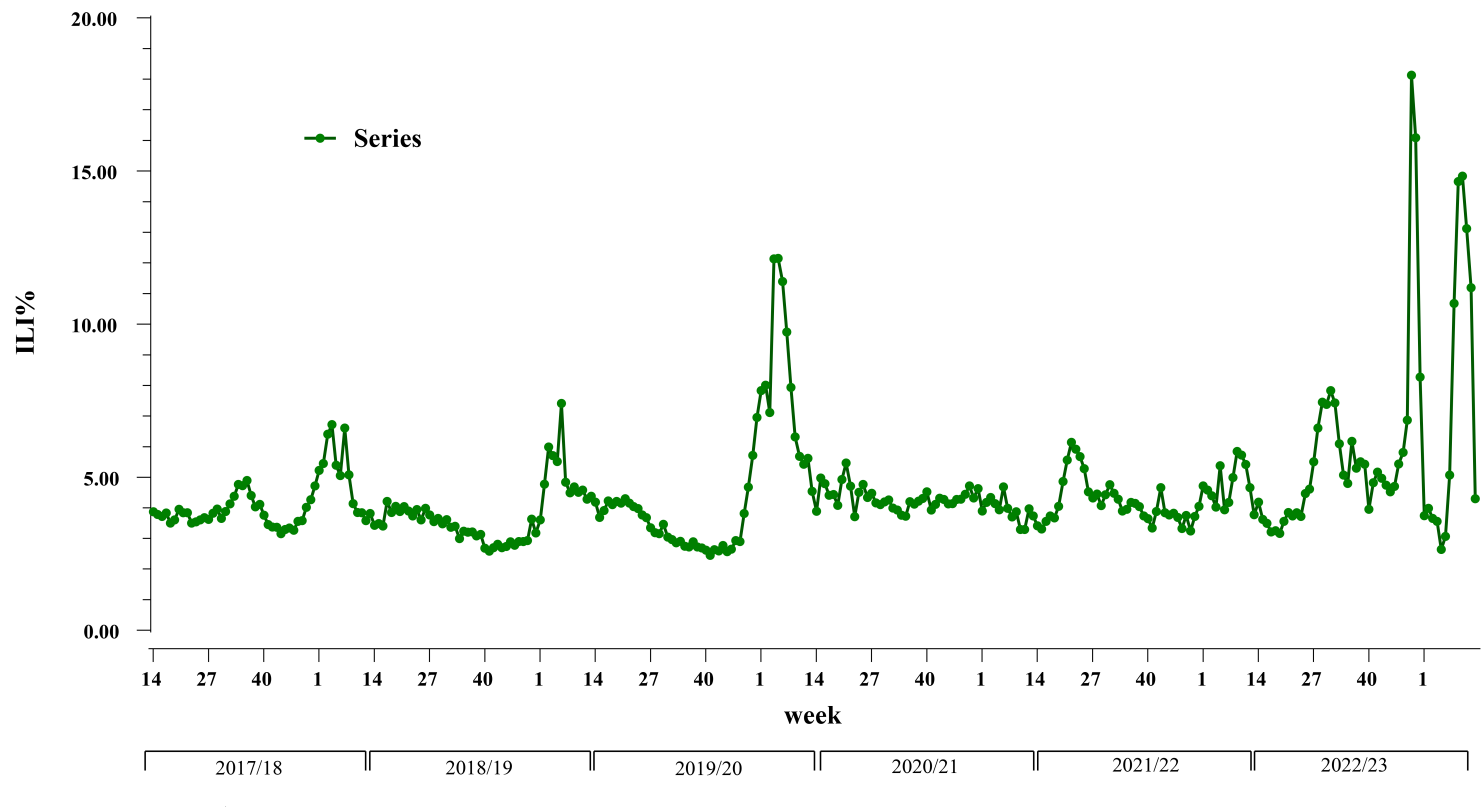
Time series of ILI%, Anhui, influenza seasons 2017/2018–2022/2023.

**Fig 2:**
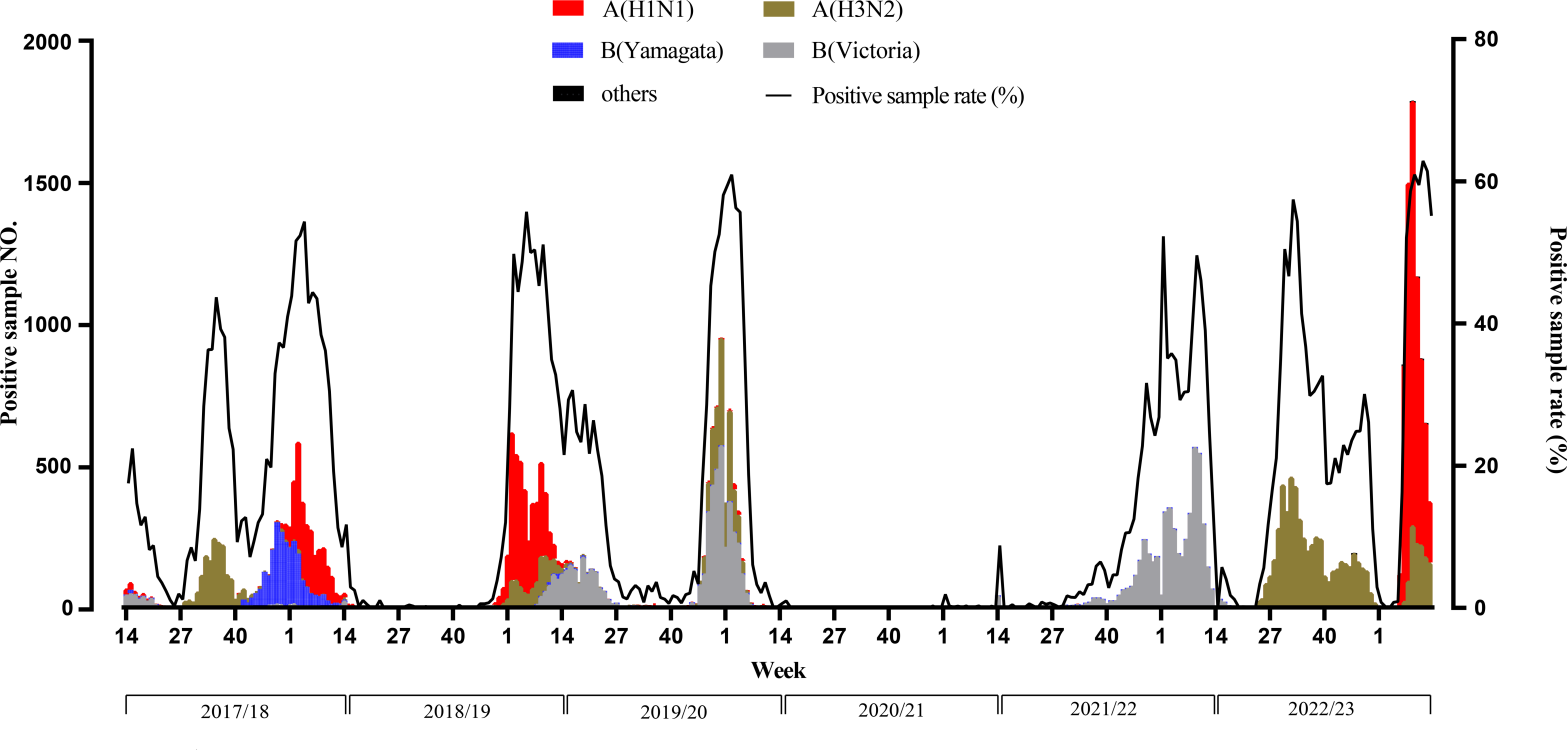
Time series of confirmed influenza cases, Anhui, influenza seasons 2017/2018–2022/2023.

The ILI% for each year, in Anhui province, from 2017/2018 to 2022/2023 was 4.30% (191148/4448211), 3.74% (177131/4730312), 4.26% (195730/4595228), 3.52% (139985/3973344), 4.33% (210176/4859347), and 6.82% (283233/4154854), respectively. The ILI% for the year 2022/2023 was higher than in previous years, and the difference was statistically significant (*χ*^2^ = 97325.74, *P* < 0.001). Notably, there were clear seasonal fluctuations in the occurrence of influenza, with two peaks during the winter-spring and summer seasons (Fig. 1). The influenza detection rates for the years 2017/2018 to 2022/2023 were 26.20% (6936/26488), 18.20% (5106/28063), 25.00% (7167/28636), 0.10% (31/27699), 15.60% (5439/34770), and 31.90% (12882/40401), respectively. The difference in detection rate between these years was statistically significant (*χ*^2^ = 11882.41, *P* < 0.001). The peaks of laboratory-confirmed cases corresponded closely to the ILI%, except for the peak in the 50th week of 2022/2023 caused by the new coronavirus. The A(H1N1) subtype was detected in 2017/2018, 2018/2019, and 2022/2023, with a more significant impact during the latter year’s outbreak (Fig. 2). The A(H3N2) subtype circulated in the first half of 2022/2023, while the B(Victoria) lineage was prevalent in the second half of 2021/22.

### Comparative analysis of epidemic intensity

There was a noticeable increase in the number of detections of the H1N1pdm09 subtype compared to other subtypes within a short period during the 2022/2023 influenza season. The last notable prevalence of H1N1pdm09 in Anhui Province occurred during the 2017/2018 to 2018/2019 influenza seasons.

We smoothed the detection rate data for six influenza seasons (2012/2013, 2013/2014–2018/2019) with H1N1pdm09 prevalence. The MEM demonstrated a good fit for the single-peak pattern. To facilitate analysis, the period from the 43rd week to the 42nd week of the following year was defined as one influenza season. The key parameter δ was determined as 2.0, with a corresponding YI of 0.866 (Table S1). The model exhibited good performance, with a sensitivity of 0.917, specificity of 0.959, and YI of 0.876 (Table S1). The predicted epidemic threshold for H1N1pdm09 was a weekly detection rate of 3.53%. A decrease in rate to 6.19% indicated the end of the epidemic season. The epidemic thresholds for moderate, high, and very high activity levels were 17.37%, 41.86%, and 61.76% per week, respectively.

**Fig 3:**
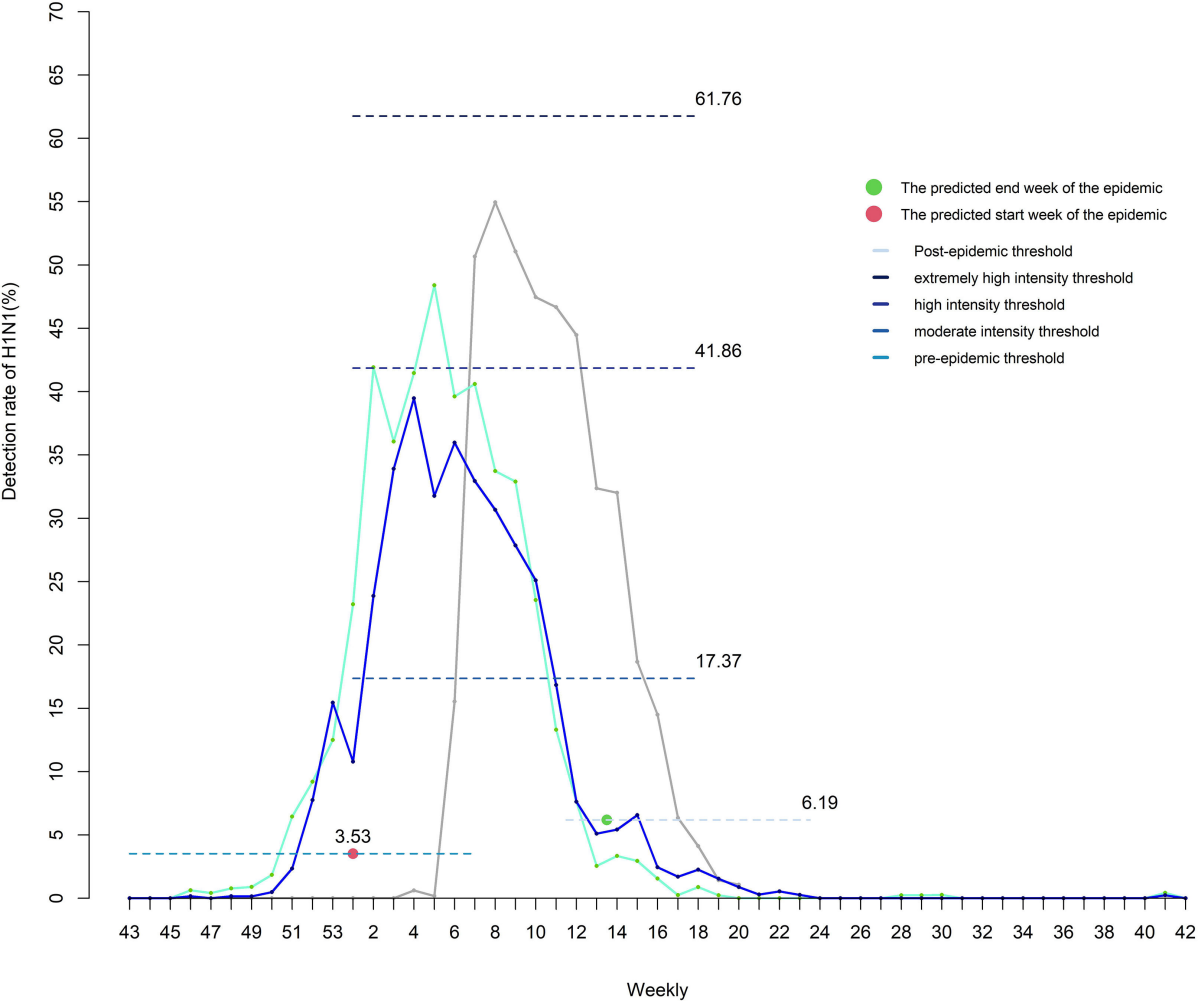
Dividing influenza seasons of 2017/2018, 2018/2019, and 2022/2023 using predictive threshold. The blue line in the graph represents the 2017/2018 influenza season, the green line represents the 2018/2019 influenza season, and the gray line represents the 2022/2023 influenza season.

To compare differences in epidemic intensity, the 3 years were segmented using predicted thresholds (Fig. 3). In the 2022/2023 year, compared to the preceding two epidemic seasons, the peak was higher, and the overall duration of the epidemic was shorter, but the duration of the high-intensity epidemic phase was longer (Table 2).

**TABLE 2:** Comparison of epidemic period lengths

Variable	Epidemic period		Moderate intensity epidemic period		High intensity epidemic period	
	Start-end (week）	During	Start-end (week）	During	Start-end (week）	During
2017/18	52—13	15	2–11	10	NA^*a*^	0
2018/19	51—13	16	1–11	9	2–6	5
2022/23	6—18	13	6–16	9	7–13	7

^*a*^ NA, not applicable.

**Fig 4:**
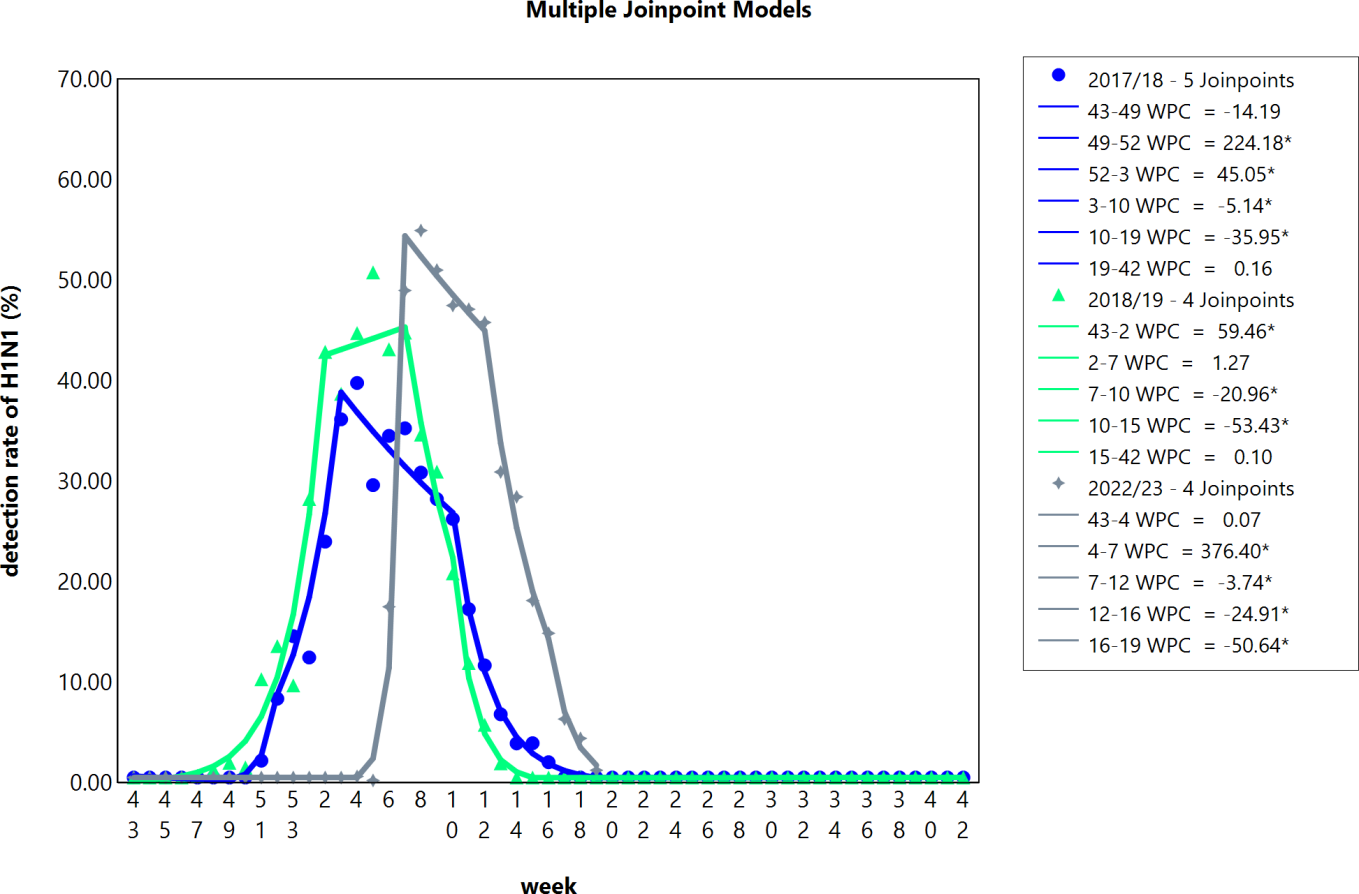
Illustrates the trend of weekly detection rates of H1N1pdm09.

Given that the weekly H1N1pdm09 incidence rates for the 3 years do not follow a normal distribution (Lilliefors corrected K-S test results provided in Table S2), a logarithmic linear model was chosen. When encountering values with zero detection rates, calculations were performed after substituting them with 0.5 (30). The results are depicted in Fig. 4. In the 2022/2023 season, compared to the preceding two A(H1N1) epidemic seasons, there was a delayed onset but a faster peak velocity and higher peak value. The A(H1N1) subtype experienced an abrupt outbreak from the fourth to the seventh week of 2023, with a WPC reaching 376.40%. During the 2017/2018 epidemic season, there was a rapid rise with a WPC of 224.18% from the 49th to the 52nd week, followed by an ascent to the peak with a WPC of 45.05% from the 52nd week to the 3rd week. The 2018/2019 epidemic season reached its peak from the 43rd to the 2nd week, with a WPC of 59.46%. Further details are provided in Table S2.

### Comparative analysis of isolates and clade classification

In 2022/2023 influenza season, the average genetic distance between the isolates from Anhui Province and the latest vaccine strains for the 2023/2024 season (A/Victoria/4897/2022, A/Wisconsin/67/2022) (31) was 1.297E-02. When compared to the previous influenza season’s vaccine strains (A/Victoria/2570/2019, A/Wisconsin/588/2019), the average genetic distance was 1.272E-02. In comparison to the viral strains from Anhui Province during the 2017/2018 influenza season, the average genetic distance was 3.112E-02. When compared to the strains from the 2018/2019 influenza season, the average genetic distance was 2.359E-02.

The majority of circulating viral strains since 2018 belong to the sub-branches of the 6B.1A lineage. For instance, according to the sequence results for 2023 strains out of 32 isolates, 30 were classified as 6B.1A.5a.2a. The remaining two belonged to 6B.1A.5a.2a.2 and 6B.1A.5a. In the 2018/2019 strains, the sub-branches 6B.1A.1, 6B.1A.2, 6B.1A.6, and 6B.1A.5a.1 revealed one strain each. Additionally, there were 2 (4.76%) of 6B.1A.3, 12 (28.58%) of 6B.1A, and 24 (57.14%) of 6B.1A.5a. During the 2017/2018 season, among the 29 isolates, one belonged to 6B.2, and in addition, there were 10 (34.48%) of 6B.1, 6 (20.69%) of 6B.1A, and 12 (41.38%) of 6B.1A.1 (Fig. S1).

### Amino acid variations in antigenic epitopes and receptor binding sites

In this study, a comparison was made between the H1N1pdm09 isolates from Anhui Province and the HA amino acid sequence of A/Michigan/45/2015. The isolates from 2017 to 2019 (71 strains) mostly exhibited mutations with S74R on the Cb epitope and S164T on the Sa epitope (60, 84.51%). On the Sb epitope, 43 strains (60.56%) had the S183P mutation. In the 2018/2019 season, the Sb epitope mutation T185I occurred in 59.52% of strains (25/42). Additionally, in the 2018/2019 season, six strains had the L161I mutation on the Sa epitope (14.29%, 6/42). There were six strains with rare mutations that were not conserved: in the 2017/2018 season, one isolate had the Sa mutation N162S, and another isolate had the Sb mutation A195T. In the 2018/2019 season, one isolate had the A195T mutation, and in two isolates, the Ca2 mutations A139T and A141T were detected. Furthermore, the isolate A/Anhui/11061/2019 had a combined mutation of D187A and Q189E on the Sb epitope. Uncommon mutations were observed at the receptor binding sites in isolates from 2017 to 2019: in the 2017/2018 and 2018/2019 seasons, each had one isolate with the mutation A195T (position 190). In the 2018/2019 season, an isolate with the E224K (position 220) mutation was also detected. However, there were no variations observed at position 130. The 2023 strains retained only the Q189E mutation at these sites, consistent with the 2020–2021 vaccine strain A/Guangdong-Maonan/SWL1536/2019.

**Fig 5:**
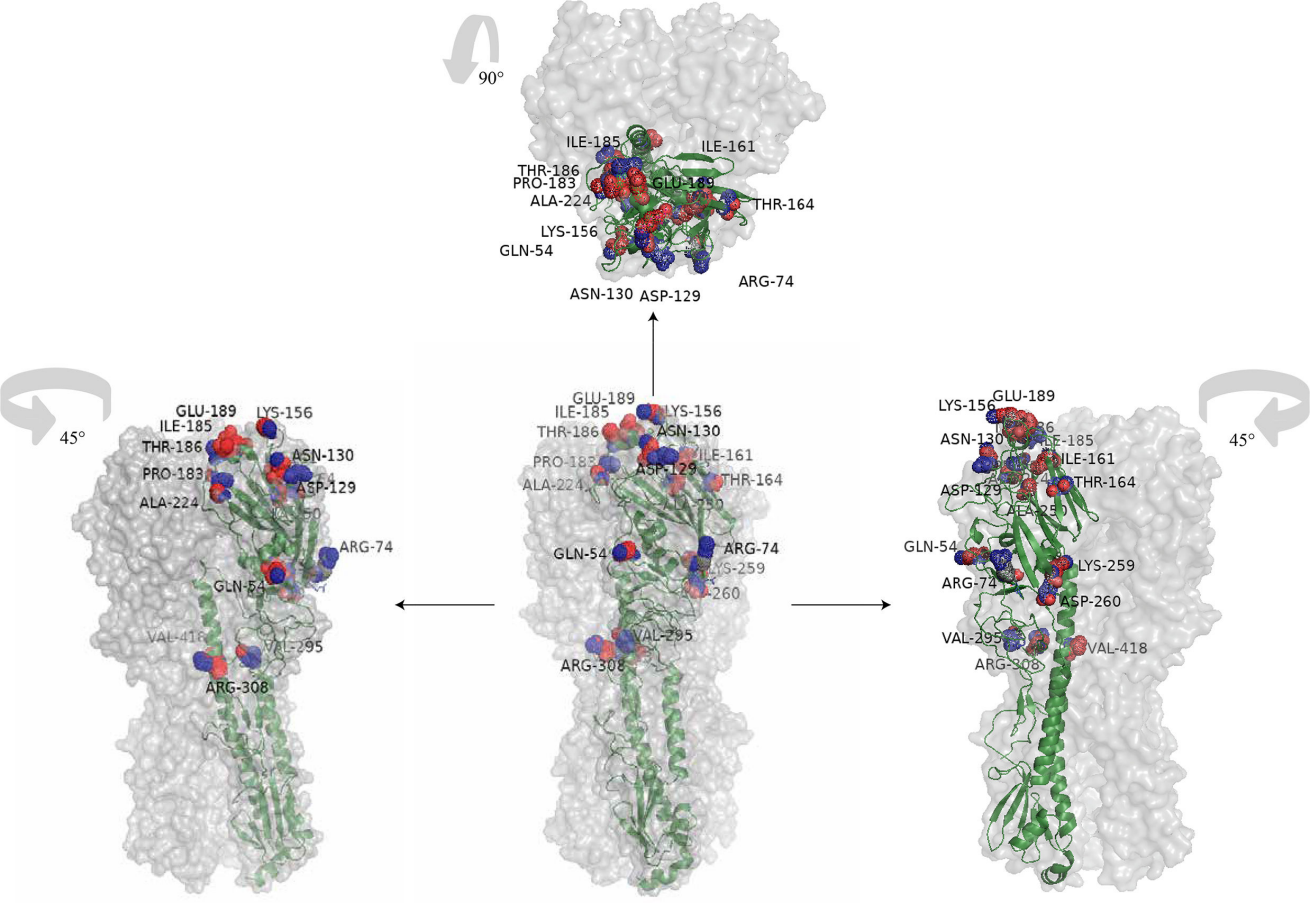
"A/Michigan/45/2015" serves as the basis for the new variant sites identified in the 2022/2023 season. The labels in the upper left/right corners indicate the rotation direction and angle. In this figure, the amino acid at position 224 is labeled as E224A, but the mutation E224S observed in the 2022/2023 year has not been indicated in the figure.

Sequence analysis for eleven strains during the 2022/2023 season (Fig. 5) revealed mutations at the following positions: S74R (Cb), S164T (Sa), S183P (Sb), and T185I (Sb), which were also frequently observed in strains isolated from 2017 to 2019. Newly emerged epitope mutations included two antigenic site mutations: N156K and L161I (Sa), A186T, and Q189E (Sb). Rare mutations included A73T on Cb, D187N on Sb, P137S, A141V, and K142R on Ca2 (Fig. S1). The Q189E mutation on the 190 helix was detected in nearly all the viral strains from the 2022/2023 influenza season. Mutations were also detected on the 220 loops, with E224A mutations in 40.62% (13/32) of strains and E224S in 56.25% (18/32) of strains, with one strain exhibiting both E224A and E222G mutations. For a comprehensive list of all detected mutation sites, please refer to Table S3.

**Fig 6:**
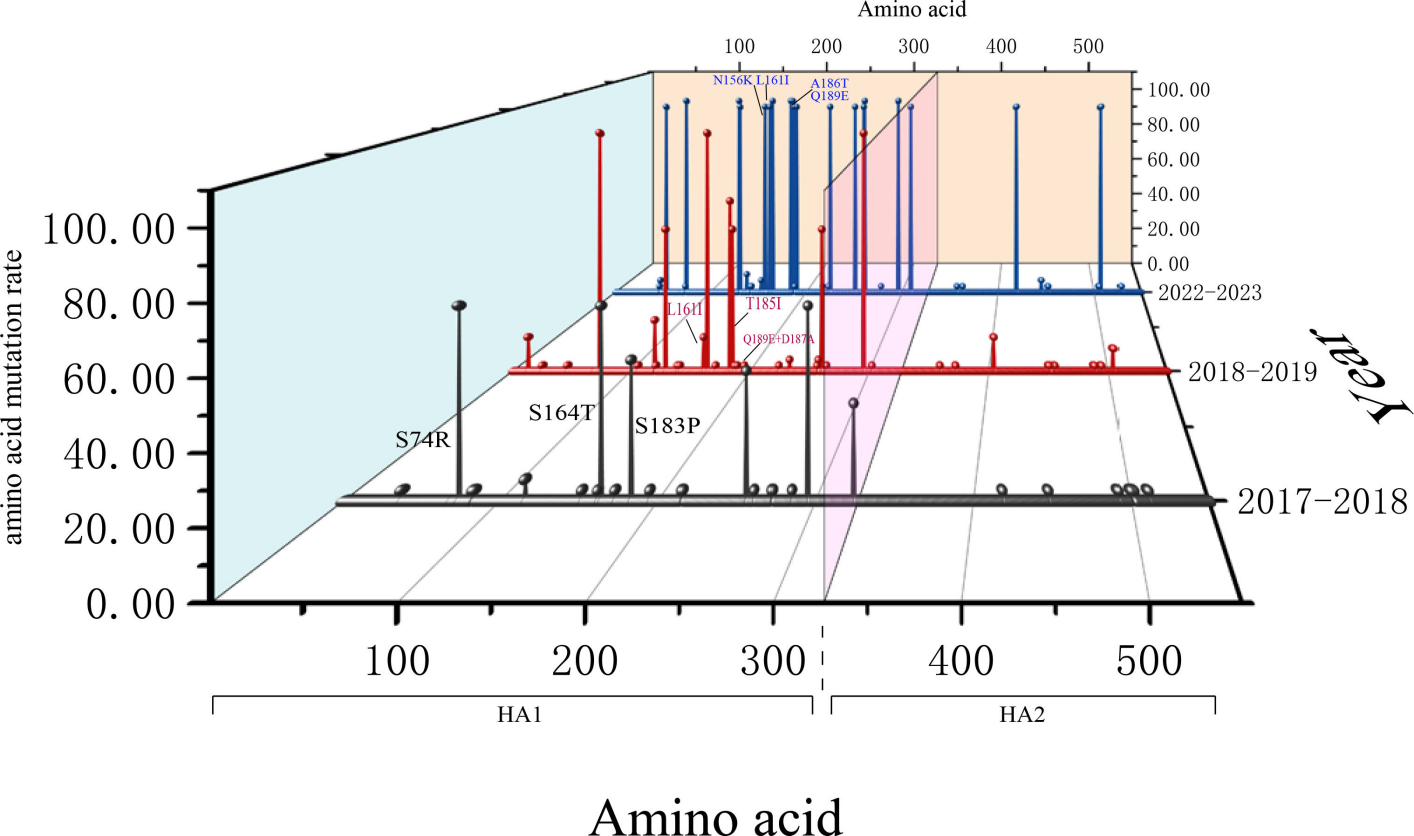
A three-dimensional plot depicting the amino acid mutation rates and conserved variable sites of H1N1pdm09 isolates from the 2017/2018, 2018/2019, and 2022/2023 influenza seasons.

The amino acid mutation sites observed over three flu seasons primarily reside in the head region of the HA1 domain. Mutations S74R, S164T, and S183P on antigenic sites have been consistently retained since the 2017/2018 flu season. Mutations L161I and T185I, which emerged during the 2018/2019 flu season, have also been consistently retained. In the 2022/2023 season, mutations N156K, L161I, A186T, and Q189E were detected in a significant number of strains. Interestingly, a mutation, Q189E, that first appeared in the 2018/2019 season was found in one strain during that season and has since been detected in all strains sequenced in the 2022/2023 season (Fig. 6).

### Prediction of glycosylation sites

The glycosylation sites for influenza strains spanning 2017/2018 to 2022/2023 were predominantly found at seven positions, and this pattern remained relatively stable since the 2017/2018 season. Only one strain, A/Anhui/SWL1195/2017, shared similarity with A/California/07/2009, and it featured six glycosylation sites due to S162 at that position. However, the emergence of the S162N mutation introduced a new glycosylation site, NQSY (162–165). Notably, N162 exhibited a dual presence of NQS (37.93%, 11/29) and the new glycosylation site NQT (62.07%, 18/29) in the 2017/2018 strains. Nonetheless, in subsequent strains, including the 2018/2019 season, it exclusively featured NQT.

### Cross-immunity protection

**Fig 7:**
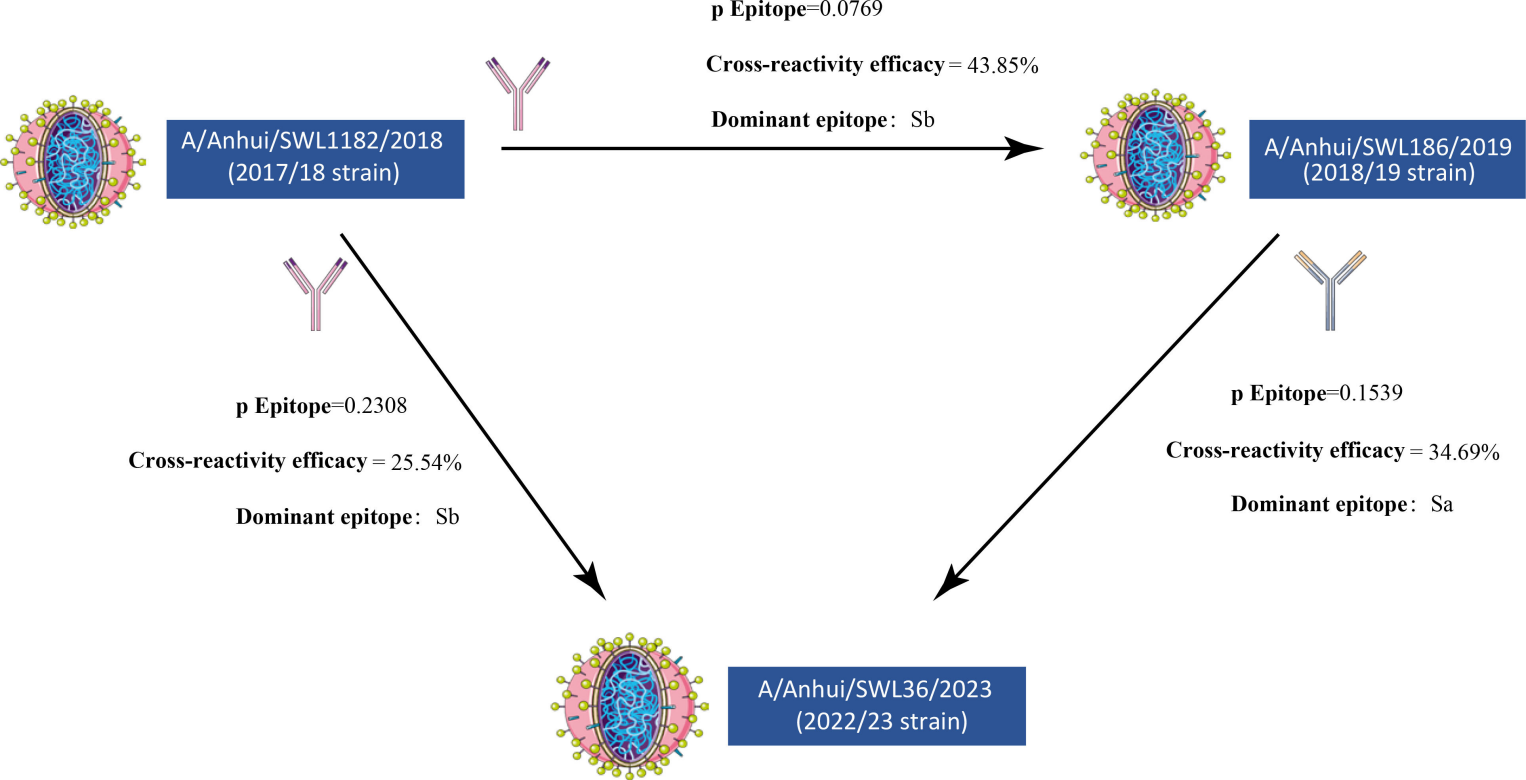
Cross-reactivity efficacy among circulating strains in the 2017/2018, 2018/2019, and 2022/2023 seasons.

To assess the cumulative impact of HA1 mutations on cross-immunity within each year, representative strains were selected from three specific seasons for evaluating antigenic distance and cross-immune effectiveness through predictive modeling. Our model projections suggest that for individuals previously exposed to A/Anhui/SWL1182/2018-like strains in the 2017/2018 season, the residual protective effectiveness against A/Anhui/SWL36/2023-like strains in the 2022/2023 season was predicted to be 48.19% (25.54% of 53.00%). Conversely, facing the A/Anhui/SWL186/2019-like strains in the subsequent season (2018/2019), the lower *P*_epitope_ value (0.0769) resulted in retained antibody protection of 82.74% (43.85% of 53.00%). For individuals previously exposed to A/Anhui/SWL186/2019-like strains in the 2018/2019 season, the effectiveness of their antibodies against the challenge of A/Anhui/SWL36/2023-like strains in the 2022/2023 season was superior to those who were solely exposed to H1N1pdm09 in the 2017/2018 season, retaining 65.45% of cross-immune efficacy (34.69% of 53.00%) (Fig. 7). This outcome contributed to the distinct rapid spread observed during the 2022/2023 season.

## DISCUSSION

Since the onset of the COVID-19 pandemic, the global spread of influenza has been significantly suppressed due to stringent public health measures and travel restrictions (5, 32). Nevertheless, from 30 January 2023, when the COVID-19 pandemic in China transitioned to a low-level phase, the H1N1pdm09 subtype in Anhui Province immediately re-emerged. Thus, conducting a retrospective comparative study on this outbreak and proposing new, target-specific control strategies for Anhui Province is instrumental in achieving cost-effective and efficient management of influenza.

In the 2022/2023 influenza season, the peak in Anhui Province was higher than in the preceding years. The peaks of ILI% and influenza positivity rate were generally consistent (except for the peak starting in week 48 of 2022/2023, which was attributed to the COVID-19 pandemic). Studies on population serum antibody levels against a specific subtype have indicated that antibody detection levels tend to decrease over time and with the continuous evolution of the virus (33, 34). This implies that the longer the interval between two successive outbreaks of a subtype, the lower the antibody levels in the population during the subsequent outbreak. Over the past 2 years, both the A(H3N2) subtype and the B(Victoria) lineage have circulated in Anhui Province. The dominance of the H1N1pdm09 subtype in the current season appears to be inevitable. The absence of the B(Yamagata) lineage since the 2017/2018 season may be linked to the faster mutation rate of influenza A compared to influenza B (35).

It should be explicitly stated that the methodology developed in this study and the proposed epidemic thresholds (designed for rapid response to emerging outbreaks) are specifically applicable to future influenza epidemics caused by the A(H1N1pdm09) subtype undergoing antigenic drift. However, these thresholds would not be valid in scenarios involving antigenic shift (which could lead to novel pandemic strains) or epidemics caused by other respiratory viruses such as respiratory syncytial viruses or coronaviruses.

The effectiveness of interventions and controls for influenza varies depending on the subtype. Therefore, neglecting the analysis and prediction of viral subtype-specific characteristics could lead to biases (36). Therefore, this study specifically focuses on establishing thresholds for the H1N1pdm09 subtype in Anhui Province and comparing the intensity of its epidemic and strain variation. This approach aids in identifying the subtype’s pattern of circulation and mutation, which in turn assists health and medical institutions in promptly implementing NPIs, planning annual vaccination schedules, and allocating essential resources effectively (19). Using prediction thresholds, based on historical data, the epidemic seasons of 2017/2018, 2018/2019, and 2022/2023 were classified. In the 2022/2023 season, the peak of the epidemic occurred during the “high” intensity phase and lasted for 7 weeks. This duration was longer compared to the 2017/2018 season (0 weeks) and the 2018/2019 season (5 weeks). Additionally, the 2022/2023 season experienced a delayed onset in the 6th week, possibly influenced by the ongoing COVID-19 pandemic impacting influenza transmission (37).

Different subtypes of influenza have varying rates of transmission. A systematic review indicated that the average generation time is reported to be between 1.9 and 5 days (36). In this study, joinpoint regression was employed to calculate and compare the WPC for the H1N1pdm09 epidemics during 2017/2018, 2018/2019, and 2022/2023 seasons. The obtained WPC values for the rapid transmission phase were 224.18%, 59.46%, and 376.40%, respectively. Subsequently, all seasons showed a “plateau phase,” characterized by a slow increase or decrease in WPC. Despite the lower WPC in the 2018/2019 season, it exhibited higher infection numbers and a longer high transmission duration compared to the 2017/2018 season. These findings might be linked to the population’s serological antibody levels. The immune system offers certain cross-protection against variant strains, but excessive antigenic distance could render previously generated memory cells ineffective (13). In the context of antigenic site mutation analysis, antibodies generated against the predominant strains of the 2017/2018 season exhibited relatively good cross-immunity to the 2018/2019 season, retaining 82.74% protection. This phenomenon might have contributed to the slower rise in WPC during the 2018/2019 season. However, the relatively lower infection numbers during the 2017/2018 season could have led to a general deficiency in antibodies within the population, resulting in a higher number of infections and prolonged transmission duration. The 2022/2023 season displayed higher WPC values compared to the previous 2 years (376.40%), coupled with a longer high transmission period attributed to the larger antigenic distance from the 2017/2018 and 2018/2019 seasons.

Through the *P*_epitope_ model, we have found that the cross-immunity antibody protective efficacy decreases over time and with virus evolution. Compared to serum antibody level detection using stratified sampling, the *P*_epitope_ model offers several advantages. First, it is time-saving and cost-effective. The model’s inference is solely based on genomic data, enabling rapid predictions upon identifying infected individuals. This facilitates prompt identification of suitable vaccine recipients, optimizes resource allocation, and requires minimal human resources. Second, it has a broad applicability. Given the time constraints, the vaccine strains provided by the WHO might not perfectly match circulating strains in different regions (12). The use of the *P*_epitope_ model for assessing vaccine efficacy allows customization of vaccine strains tailored to specific locales.

In the 2022/2023 flu season, rare mutations like A73T, P137S, A141V, K142R, and D187N were detected, some of which have never been observed on antigenic sites before. These rare mutations have the potential to contribute to future outbreaks. It’s important to note that the widely observed variation sites S74R, S164T, and S183P in this study do not appear to be associated with disease severity (38). In the 2022/2023 influenza season, isolated strains from Anhui Province exhibited mutations at the E224S sites. These mutations have not been observed in previous Anhui circulating strains or the latest vaccine strains. The impact of these mutations on virus replication and transmission warrants further investigation. Additionally, the combined mutations N156K, A186T, and Q189E were observed. A study conducted in Japan on A(H1N1) pdm09 virus characteristics from 2018 to 2020 indicated a detection rate of 10.1% for N156K in the 2019–2020 season, while the mutations D187A and Q189E were detected at a rate of 89.4%. Experimental evidence suggests that N156K could impact antigenicity, and the combined D187A and Q189E mutations at the receptor-binding site hinder recognition by ferret antisera and have also been observed in humans (39).

According to sequences registered on Nextstrain (https://nextstrain.org/flu/seasonal/h1n1pdm/ha/2y), it is inferred that the 6B.1A.5a.1 (D187A + Q189E) subclade possibly emerged on 24 December 2018, and the 6B.1A.5a.2a (Q189E + A186T) subclade is estimated to have emerged on 3 September 2020. The appearance of new local subclades often triggers a new round of outbreaks. Therefore, this study proposes a practical control strategy called the “surveillance threshold and genome assessment to measures (STGAM).” When the detection rate of each subtype reaches the threshold set by the MEM model (e.g., 3.53% for Anhui Province’s H1N1pdm09), the first detecting healthcare institution should promptly sequence and compare the virus. If a new genotype not previously circulating locally is detected, timely epidemic forecasts should be provided to the public. Intervention strategies can be implemented using the *P*_epitope_ model, with options to either implement NPIs on a larger scale or promote vaccine uptake based on compatibility between newly detected circulating strains and vaccine strains. This strategy helps to ensure that there is no disruption of socio-economic activities and peoples’ livelihoods while effectively containing the spread of the epidemic.

However, there are a few limitations to acknowledge: (i) the prediction of the start of the epidemic is based on mathematical models using historical data and has not been validated with laboratory testing of pre-epidemic population serum levels; (ii) the study calculates MEM-based epidemic thresholds specifically for Anhui Province’s H1N1pdm09. Similar thresholds will be calculated for other subtypes to make the “STGAM” applicable to different circulating strains in Anhui Province; (iii) due to the lack of annual vaccine effectiveness case-control studies in Anhui Province, the parameters used for the *P*_epitope_ model are derived from epidemiological literature from different times and regions. Although these parameters have been applied in Thailand and Wuhan, China (28, 40), it is essential for China to conduct long-term vaccine effectiveness case-control studies to obtain more suitable parameters for the local population. Continuous analysis of monitoring data and virus mutations can aid in refining more objective predictive models. This process is crucial for implementing effective and cost-efficient prevention and control measures, ultimately mitigating the public health threats posed by influenza viruses.

### Conclusions

The results above indicate that the 2022/2023 H1N1pdm09 influenza season exhibited a more aggressive nature, with higher transmission rates, increased epidemic intensity, and a larger number of infections compared to the preceding two seasons. Furthermore, we explored the molecular-level factors contributing to this outbreak. Finally, we propose a targeted intervention strategy known as STGAM, which involves updating the early warning threshold annually based on surveillance data. Subsequently, depending on sequencing results and immune efficacy, prioritized selection of NPIs or vaccine campaigns in public spaces is recommended. In cases of suboptimal vaccine efficacy, prompt adjustments to vaccine strain components are advised.

## Data Availability

All data generated or analyzed during this study are included in this published article. Besides, any additional data/files may be obtained from the corresponding author on reasonable request.

## SUPPLEMENTAL MATERIAL

Fig. S1 spectrum.02218-24-s0001.tif Phylogenetic tree of influenza A H1N I pdm09 virus HA nucleotide sequences in Anhui province.

Table S1 spectrum.02218-24-s0002.xlsx The good performance of the model.

Table S2 spectrum.02218-24-s0003.xlsx The key parameter δ of the model.

Table S3 spectrum.02218-24-s0004.xlsx Lilliefors corrected K-S test results.

ASM does not own the copyrights to Supplemental Material that may be linked to, or accessed through, an article. The authors have granted ASM a non-exclusive, world-wide license to publish the Supplemental Material files. Please contact the corresponding author directly for reuse.
